# Association between insulin resistance with UCP2 -866G/A, UCP2 45BP INS/DEL, UCP3 -55C/T, GHSR1A RS2922126, GHSR1A RS509035 and PRO12ALA PPARΓ2 gene polymorphisms in obese female adolescents in Yogyakarta, Indonesia

**DOI:** 10.1186/1687-9856-2013-S1-O36

**Published:** 2013-10-03

**Authors:** Rina Susilowati, Dian Eurike Septyaningtrias, Cut Gina Inggriyani, Harry Freitag Luglio Muhammad, Madarina Julia

**Affiliations:** 1 Department of Histology and Cell Biology, Faculty of Medicine Universitas Gadjah Mada Jalan Bulaksumur, Indonesia; 2 Department of Health Nutrition, Faculty of Medicine Universitas Gadjah Mada Jalan Bulaksumur, Indonesia; 3 Department of Child Health Faculty of Medicine Universitas Gadjah Mada Jalan Bulaksumur, Indonesia

## Aims

The aim of this study was to analyze the association between polymorphism of several genes encoded the uncoupling proteins (UCPs), ghrelin receptors (GHSRs) and peroxisome proliferator-activated receptor gamma (PPARγ) with insulin resistance in obese female adolescents in Yogyakarta, Indonesia.

## Methods

Screening for obesity using CDC 2000 criteria was done in 2121 female adolescents aged 13-14 years old in Yogyakarta. BMI > 95^th^ percentile was considered as obese. Among the obese subjects, 78 agreed to be enrolled for this study. HOMA-IR > 3.16 was used to determine the insulin resistance status. DNA was isolated from peripheral blood and UCP2 -866 G/A, UCP3 -55C/T, GHSR1a rs2922126, GHSR1a rs509035 and Pro12Ala PPARγ2 genotypes were analyzed by PCR-RFLP. UCP2 45bp ins/del genotype was analyzed by PCR.

## Results

Among the 78 obese adolescent girls, 44 (56.4%) were at insulin resistance state. All subjects had Pro12Pro PPARγ2 and del/del UCP2 genotype. Compared to the other polymorphisms analyzed in this study, the AA genotype and the A allele of UCP2 -866 G/A polymorphism was found to have highest association with insulin resistance state (OR: 2.75; 95% CI 0.65 – 11.62; p=0.17 for AA genotype; OR 1.50; 95%; CI 0.79 – 2.83; p=0.22 for A allele). In UCP3 -55C/T polymorphism, TT genotype also showed positive statistically not significant association with insulin resistance (OR 2.32; 95% CI 0.38 – 14.12; p=0.36), so did T allele (OR 1.30; 95% CI 0.67 – 2.50; p=0.45). Genotyping of the Ghrelin receptor gene showed also non significant association with insulin resistance, i.e. the AA genotype (OR 2.25 ;95% CI 0.21–24.4; p=0.63) and A allele (OR 1.05; 95% CI 0.54 – 2.05; p=0.89) of the GHSR1a rs509035 polymorphism as well as AA genotype (OR 2.03; 95% CI 0.54 – 7.57; p=0.28) and A allele (OR 1.36 ; 95% CI 0.72 – 2.58; p=0.34) of the of the GHSR1a rs2922126 polymorphism.

## Conclusion

We observed not statistically significant association between gene polymorphism of UCP2 -866G/A, UCP3 -55C/T, GHSR1a rs509035, GHSR1a rs2922126 and the incidence of insulin resistance in obese female adolescent in Yogyakarta Indonesia.

